# The Effects of Natural Iron Fertilisation on Deep-Sea Ecology: The Crozet Plateau, Southern Indian Ocean

**DOI:** 10.1371/journal.pone.0020697

**Published:** 2011-06-14

**Authors:** George A. Wolff, David S. M. Billett, Brian J. Bett, Jens Holtvoeth, Tania FitzGeorge-Balfour, Elizabeth H. Fisher, Ian Cross, Roger Shannon, Ian Salter, Ben Boorman, Nicola J. King, Alan Jamieson, Frédéric Chaillan

**Affiliations:** 1 School of Environmental Sciences, University of Liverpool, Liverpool, United Kingdom; 2 National Oceanography Centre, University of Southampton Waterfront Campus, Southampton, United Kingdom; 3 Oceanlab, University of Aberdeen, Newburgh, United Kingdom; University of California Merced, United States of America

## Abstract

The addition of iron to high-nutrient low-chlorophyll (HNLC) oceanic waters stimulates phytoplankton, leading to greater primary production. Large-scale artificial ocean iron fertilization (OIF) has been proposed as a means of mitigating anthropogenic atmospheric CO_2_, but its impacts on ocean ecosystems below the photic zone are unknown. Natural OIF, through the addition of iron leached from volcanic islands, has been shown to enhance primary productivity and carbon export and so can be used to study the effects of OIF on life in the ocean. We compared two closely-located deep-sea sites (∼400 km apart and both at ∼4200 m water depth) to the East (naturally iron fertilized; +Fe) and South (HNLC) of the Crozet Islands in the southern Indian Ocean. Our results suggest that long-term geo-engineering of surface oceanic waters via artificial OIF would lead to significant changes in deep-sea ecosystems. We found that the +Fe area had greater supplies of organic matter inputs to the seafloor, including polyunsaturated fatty acid and carotenoid nutrients. The +Fe site also had greater densities and biomasses of large deep-sea animals with lower levels of evenness in community structuring. The species composition was also very different, with the +Fe site showing similarities to eutrophic sites in other ocean basins. Moreover, major differences occurred in the taxa at the +Fe and HNLC sites revealing the crucial role that surface oceanic conditions play in changing and structuring deep-sea benthic communities.

## Introduction

Artificial ocean iron fertilization (OIF) of high nutrient low chlorophyll (HNLC) oceanic waters and subsequent carbon drawdown [1] could provide a contribution to mitigation of anthropogenic atmospheric CO_2_ [2], [3], [4]. Artificial OIF involves the seeding of surface HNLC waters with iron, for example as FeSO_4_ [5]. However, the impacts of large-scale manipulation of oceanic waters on pelagic and benthic ecosystems are unknown [1], [6]. While spatial and temporal variations in total biomass and gross taxon composition of deep-sea metazoan macrofauna and megafauna appear to reflect changes in upper ocean conditions [7], [8], [9], the relationship between species distributions and surface ocean productivity is not understood. This information is vital in assessing the consequences of large-scale manipulations of surface ocean productivity and carbon sequestration by artificial OIF [10].

**Figure 1:**
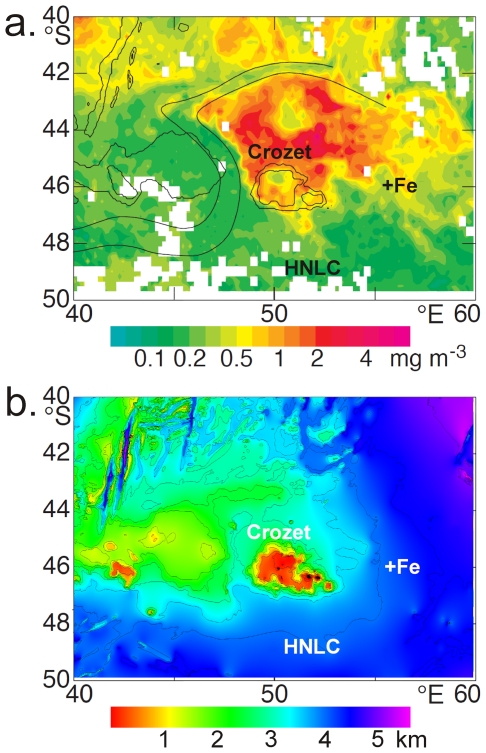
Location maps of the +Fe and HNLC sites with respect to: **a.** monthly (October 1998) SeaWiFS chlorophyll (mg m^−3^) distribution, showing a typical spring bloom to the north of the Crozet Plateau (adapted from Ref. 15), with the meander of the Sub-Antarctic Front indicated by parallel black lines. **b.** Water depth (km) in the corresponding Crozet region (derived from the General Bathymetric Chart of the Oceans, Centenary Edition).

The Southern Ocean is the largest HNLC region on Earth. Within this expanse, there are hotspots of primary productivity during the Austral spring and summer. These occur in areas of natural OIF, through the entrainment of dissolved iron leached from isolated oceanic islands, such as at the Kerguelen and Crozet Plateaus [11], [12]. The chemical form of natural iron differs from FeSO_4_; while its precise chemistry is not well understood, a large fraction is likely to be complexed by humic materials or other ligands [13], [14]. Fe enrichment over the light-limited winter period to the north and east of the Crozet Plateau (48°S 56°E) in the Southern Indian Ocean, leads to a seasonal phytoplankton bloom when solar irradiance increases and the critical depth exceeds that of the mixed layer due to the onset of stratification in the spring. The spatial extent of the bloom is controlled by a semi-permanent meander in the Sub-Antarctic Front (Fig. 1a), while the water circulation is such that south of Crozet HNLC conditions prevail [15]. SeaWIFs images confirm that the hotspots of primary production stimulated by natural iron fertilization occur consistently from year to year [12], [16] and it is likely that these seasonal high productivity regions have persisted throughout the Holocene [17].

The Crozet Plateau provides an ideal opportunity to study the impact of nutrient enrichment in surface waters on the ecology of the deep-ocean floor. Studies of upper ocean biogeochemistry in the Austral summer of 2004/2005 [11], [12] confirmed enhanced primary productivity in surface waters to the north and east of the Crozet Plateau stimulated by iron enrichment. Sediment traps deployed over periods of ∼12 months from December 2004 to January 2006 [12], [18] indicated enhanced export of carbon to the deep ocean, north and east of the Crozet Islands [11], [12]; the annual carbon flux at ∼3000 m water depth in iron-enriched waters was ∼2.5× greater than the flux in HNLC waters south of the Plateau [12] during this period.

We undertook an intensive research programme during the Austral summer of 2005/2006 around the Crozet Islands in the Southern Ocean [19], to test for the first time our hypothesis that abyssal benthic ecology is influenced by natural OIF. We chose two sites, +Fe and HNLC (460 km apart), at the same water depth (∼4200 m) and in similar geomorphological, hydrographic and sedimentary settings with no physical barrier between them (Fig. 1b) [20]. The two sites are almost identical in their environmental characteristics apart from the productivity of overlying surface waters. The absence of geomorphological and hydrographic barriers between the two sites ensured that there are no constraints on the dispersal of fauna, other than that which might be related to surface water productivity. We measured fluxes of organic matter in the water column, with a specific aim of assessing the composition and quality of the sinking particulate organic matter. We investigated the megabenthos (>4 cm and large enough to be seen in bottom photographs; [21]) by means of trawls and video transects. These fauna, in particular holothurians, are a good group to use to investigate the influence of productivity from surface waters because they feed almost exclusively on freshly deposited detritus on the seafloor [22]. The holothurians dominate the deep-ocean megabenthos, sometimes accounting for up to 95% of individuals [22]. The ophiuroids are another important group, which feed on a variety of substrates [23]. Macrofauna are not included, because we were unable to collect sufficient samples, while results for the meiofauna and fish are reported elsewhere [19], [20].

## Materials and Methods

A comprehensive account of sampling activities is given in the RRS *Discovery* cruise 300 Cruise Report [19] together with a narrative and full station list. We summarise relevant sampling activities in Table 1.

**Table 1:** Details of selected scientific deployments at +Fe and HNLC sites around the Crozet Plateau.

Site	Discovery Station Number	Latitude (S)	Longitude(E)	Date	Depth (m)	Gear	Sampling type
+Fe	15773#6	45°50.43′	56°06.16′	12/12/2005	4157	ROBIO	Bottom video/stills
+Fe	15773#8	45°43.06′	56°32.16′	12/12/2005	4258–4290	Trawl	Invertebrate megabenthos
+Fe	13773#9	45° 50.45′	56°24.78′	13/12/2005	4200	WASP	Bottom video/stills
+Fe	15773#13	45°50.47′	56°06.42′	14/12/2005	4162	ROBIO	Bottom stills
+Fe	15773#17	45°43.47′	56°36.66′	15/12/2005	4301–4283	Trawl	Invertebrate megabenthos
+Fe	15773#20	45°53.34′	56°24.24′	15/12/2005	4189	Megacorer	Surface sediments
+Fe	15773#21	45°53.67′	56°24.40′	15/12/2005	4193	Megacorer	Surface sediments
+Fe	15773#23	45°40.05′	56°35.27′	16/12/2005	4269–4275	Trawl	Invertebrate megabenthos
+Fe	15773#25	45°55.07′	56°27.95′	17/12/2005	4203	Megacorer	Surface sediments
+Fe	15773#26	45°54.10′	56°25.42′	17/12/2005	4188	SAPs	Suspended POM
+Fe	15773#28	45°53.81′	56°25.03′	18/12/2005	4191	Megacorer	Surface sediments
+Fe	15773#30	45°00.00′	56°05.00′	26/12/2004–20/12/2005	3195	Sediment trap mooring	Sinking POM flux
+Fe	15773#31	45°53.56′	56°25.77′	20/12/2005	4200	Megacorer	Surface sediments
+Fe	15773#32	45°40.45′	56°33.70′	20/12/2005	4267–4270	Trawl	Invertebrate megabenthos
+Fe	15773#42	45°53.88′	56°25.38′	24/12/2005	4194–4196	WASP	Bottom video/stills
HNLC	15775#3	49°03.65′	51°14.21′	27/12/2005	4202	Megacorer	Surface sediments
HNLC	15775#4	48°56.21′	51°03.90′	27/12/2005	4182–4195	Trawl	Invertebrate megabenthos
HNLC	15775#10	49°03.99′	51°14.02′	29/12/2005	4204	Megacorer	Surface sediments
HNLC	15775#13	49°01.15′	51°04.52′	29/12/2005	4187–4191	Trawl	Invertebrate megabenthos
HNLC	15775#15	49°11.41′	51°09.56′	30/12/2005	4221	SAPs	Suspended POM
HNLC	15775#19	49°04.59′	51°13.49′	31/12/2005	4202	Megacorer	Surface sediments
HNLC	15775#23	49°00.03′	51°30.59′	03/01/2005–03/01/2006	3183	Sediment trap mooring	Sinking POM flux
HNLC	15775#26	49°04.63′	51°11.48′	03/01/2006	4169–4193	WASP	Bottom video/stills
HNLC	15775#37	49°01.88′	51°14.10′	05/01/2006	4192	Megacorer	Surface sediments

### 2.1. Collection of samples

#### 2.1.1. Fauna

Sampling of megafauna was undertaken with a semi-balloon otter trawl (OTSB 14) [24] with a wing-end spread of 8.6 m. While being towed on the bottom at nominal speeds of 0.75 ms^−1^, the height of the net from the footrope to the headline is ∼1.5 m. Contact of the net with the seafloor, and hence estimates of the area fished, was assessed by changes in wire tension during trawling operations. Catches were immediately sorted into major taxa and fresh wet weight biomass determined by batch weighing using a marine bench balance. Specimens for biochemical analysis were transferred to a cold room (4°C) and immediately dissected and samples frozen (−70°C). Other material was subsequently fixed in 5% borax-buffered seawater formaldehyde and later (3–5 days) transferred to 80% Industrial Denatured Alcohol. Comparisons of relative biomass composition between the two Crozet sites and with a site on the Porcupine Abyssal Plain (north-eastern Atlantic Ocean) were carried out by cluster analysis using the PRIMER 6 software [25]. Data were compiled for major holothurian families (Synallactidae, Elpidiidae, Psychropotidae, Deimatidae), combined ‘other holothurians’ and combined ‘other invertebrates’, standardised (converted to proportion of total catch), Bray-Curtis similarities calculated, and a group-average clustering strategy employed to produce a dendrogram.

#### 2.1.2. Sea-bed photography

The Wide-Angle Seabed Photography system (WASP) was used to provide sea floor photographs. WASP is a self-contained, off-bottom, towed camera vehicle that provides still and video footage of the seabed fitted with: OSIL (Ocean Scientific International Ltd, UK) Mk7 (stills) camera, OSIL 1200 J flash gun, NOC OceanCam6000V (digital video) camera, 2×250 W Deepsea Power and Light (DSPL) video lamps, 3×DSPL 24 V batteries, Simrad Mesotech 200 kHz altimeter, and a NOC acoustic telemetry system (10 kHz). The vehicle was operated at ∼3 m above the seabed. The still and video cameras were both automatically activated by the altimeter when the range to the seabed was <10 m. For all deployments made during the cruise, the still camera was loaded with Kodak Vision 250D colour negative 35 mm film (c. 40 m loads) and the video camera loaded with a 65-minute MiniDV tape.

Other photographs were obtained using the RObust BIOdiversity lander (ROBIO) [26]. This is a baited time-lapse camera system used to assess the abundance and diversity of scavenging fish fauna. It was equipped with a 3 megapixel digital camera (Kongsberg OE-218) and flash-gun; the time-lapse interval was pre-programmed and images were recorded internally. ROBIO was deployed with the instrument frame tethered 2 m above the seabed, between 100 kg of ballast below and floatation above.

#### 2.1.3 Particulate organic matter

Sinking POM was collected using deep-sea sediment traps. These were deployed in December 2004 and recovered in December 2005 for the +Fe site and deployed in January 2005 and recovered in January 2006 for the HNLC site (Table 1). All sediment traps were funnels (0.5 m^2^ surface area) with a baffled aperture and a narrow opening at the bottom leading to the sampling rosette (McLane INC. Parflux ∼21cup). Sample bottles were filled with buffered preservative solution prepared according to JGOFS protocols by adding 100 g of analytical grade NaCl to 19 L of unfiltered deep (>2000 m) seawater from the trap deployment locations. 1 L of formaldehyde (AnalR® grade, VWR International) was buffered to pH 8.6 with sodium tetraborate and added to the 19 L of hypersaline seawater solution and left to stand for a period of 1 day. On recovery samples were filtered through a 1 mm Nitex mesh. Swimmers were removed from the <1 mm fraction. The >1 mm size fraction was comprised exclusively of large swimmers. Some cups were contaminated by fish (*Notolepis coatsi*) feeding on the sinking material. All fish debris was picked out by hand. All chemical analyses were performed on the <1 mm fraction. The 250 mL samples were split into 8 aliquots of equal volume using a rotary splitter. According to sample mass, 1–4 of these eighths were filtered onto glass-fibre filters (Whatman GF/F, 47 mm, 0.7 µm pore size). The samples and filters were subsequently rinsed with a 0.56 M ammonium formate solution (pH 7) to remove excess sea-salt and formalin. The filters were then freeze-dried and stored (−20°C) prior to analysis.

Suspended particulate matter was collected close to the sea floor using large-volume stand-alone pumps (SAPs; Challenger Oceanic). The particulate material was collected on two pre-ashed (450°C; 4 h) filters (293 mm diameter; GF/F). The filters were wrapped in dichloromethane-rinsed foil, stored for the duration of the cruise (−70°C), freeze-dried on return, and then stored until analysis (−20°C).

#### 2.1.4 Sediments

Sediments were collected using a Bowers-Connelly Megacorer [20]. On recovery, all cores were immediately taken to a constant temperature laboratory (4°C) for further processing. For chemical analyses of surface sediments, small cores (50 mm diameter) were sliced and the top 5 mm section was packed in clean (400°C; 12 h) aluminum foil and stored in Petri dishes (−70°C), before being freeze-dried on return and stored until analysis (−20°C).

### 2.2 Chemical analyses

#### 2.2.1. Total organic carbon and nitrogen

Elemental analyses of the sediments were carried out as described by Kiriakoulakis et al. [27]. Briefly, organic carbon contents were determined using aliquots of freeze-dried sediments following de-carbonation by the acid HCl vapour. Determination of total carbon and nitrogen was performed on the non-decarbonated sediments. All analyses were carried out in duplicate using a CE-Instruments NC 2500 CHN analyser. Reproducibility of the analytical technique was <±10%. Analytical precision calculated as the relative standard deviation from the mean for the standard (n = 25) is ±0.07 and ±0.01% for C and N, respectively.

#### 2.2.2. Lipids

Procedures for lipid analyses of both filters and sediments have been described elsewhere [27]. Briefly, separate aliquots of freeze-dried sediment (1–5 g) or filter material (∼1 g) were spiked with an internal standard (5α(H)-cholestane), sonicated (filters; 45 min; dichloromethane∶methanol 9∶1), methylated (methanolic acetyl chloride) and silylated (bis-trimethylsilyltrifluoroacetamide; 1% trimethylsilane chloride; 30–50 µL; 40°C; 0.5–1 h). GC-MS analyses were carried out using a Trace 2000 Series GC (on-column injector; fused high temperature silica column, 60 m×0.25 mm i.d.; 5% phenyl/95% methyl polysiloxane equivalent phase, 0.1 µm film thickness, DB5-HT, J&W carrier gas helium at 1.6 mL min^−1^). Typically, the oven temperature was programmed from 60°C to 170°C at 6°C min^−1^ after 1 minute, and then to 315°C at 2.5°C min^−1^ and held (10 min). The column was fed directly into the EI source of a Thermoquest Finnigan TSQ 7000 mass spectrometer. Typical operating conditions were: ionisation potential 70 eV; source temperature 215°C; trap current 300 µA. Mass data were collected at a resolution of 600, cycling every s from 50–600 Thompsons and were processed using Xcalibur software. Compounds were identified either by comparison of their mass spectra and relative retention indices with those available in the literature and/or by comparison with authentic standards. Quantitative data were calculated by comparison of peak areas of the internal standard with those of the compounds of interest, using the total ion current (TIC) chromatogram. The relative response factors of the analytes were determined individually for 36 representative fatty acids, sterols and alkenones using authentic standards. Response factors for analytes where standards were unavailable were assumed to be identical to those of available compounds of the same class. Reproducibility of the lipid extraction procedure and analysis are reported by Jeffreys et al. [28].

#### 2.2.3. Pigments

In order to evaluate the gonad index and their biochemistry of the holothurians, samples of their ovarian tissue were dissected, freeze-dried and weighed. Pigments (carotenoids) were extracted in 3 mL of 90% HPLC grade acetone/water. Samples were ultrasonicated for 30 seconds then centrifuged for 10 mins at 3000 rpm. The extract was passed through a (0.2 µm) Nyalo membrane filter (Gelman) prior to analysis. Samples were transferred to amber vials and loaded into a chilled (0°C) HPLC autosampler tray. Aliquots of sample (500 µL) were mixed with 1 M ammonium acetate (500 µL) and 100 µL of this mixture injected onto the HPLC column. Pigment samples were separated using ion paired reverse phase HPLC according to Barlow et al. [29]. The HPLC was controlled by the ChromQuest software system. It consisted of either a Perkin Elmer C18 column [30] or Perkin Elmer C8 column [29], Thermoseparation HPLC system with an online vacuum degasser, a dual solvent pump (P2000), autosampler (AS3000), a UV photodiode array detector (UV6000) and a Spectra System fluorescence detector (FL3000). Chlorophylls and carotenoids were detected by absorbance at 440 nm; pheopigments were monitored with the fluorescence detector using excitation and emission wavelengths of 410 and 670 nm, respectively. Pigments were identified by comparison of relative retention times with pigment standards. Supporting identification was gained by comparison of spectral data with known standards as well as by reference to the Jeffrey et al. [31]. Response factors for each of the pigments were calculated by plotting concentrations of the standards against peak area. Reproducibility of the analytical technique was better than ±10% and the analytical precision <±5%.

## Results

**Figure 2:**
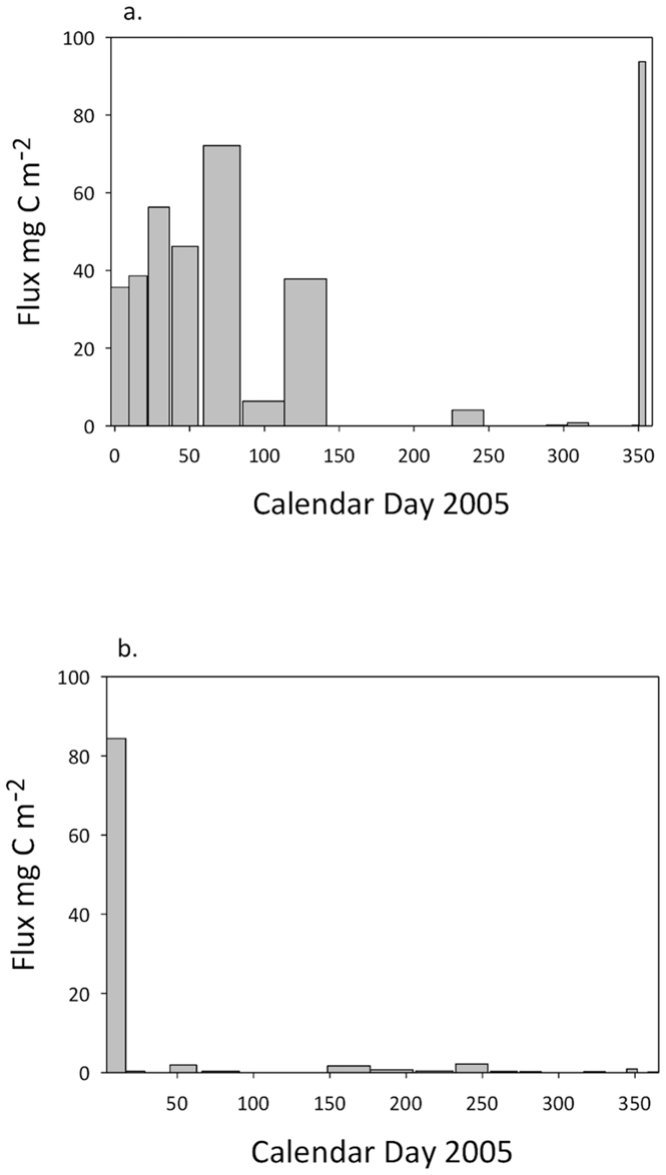
Carbon fluxes determined over a period of ∼12 months in 2005 at the Crozet Plateau (see also Ref. 12): a. the +Fe site at 3183 m water depth b. the HNLC site at 3195 m water depth. Both ∼1000 m above the seabed. Bar widths are proportional to cup opening times of the Parflux sediment traps. Bar heights represent the material supplied over the period of cup opening.

**Figure 3:**
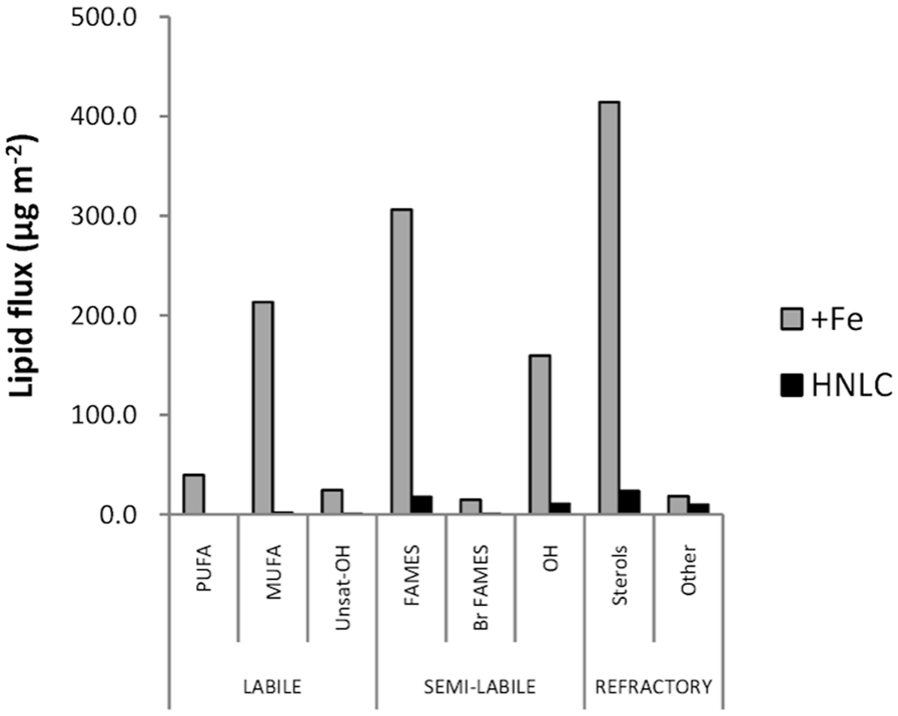
Lipid fluxes at the Crozet abyssal locations, +Fe and HNLC at 3195 and 3183 m water depth in 2005. **Labile lipids**: Polyunsaturated fatty acids (PUFA), monounsaturated fatty acids (MUFA) and unsaturated alkanols (U-ALK); **Semi-labile lipids**: Saturated fatty acids (FA), branched fatty acids (Br-FA) and *n*-alkanols (ALK); **Refractory lipids**: Sterols (STER) and other lipids including branched alkanols, triterpenoids, steroidal ketones, phytol derivatives and alkenones (OLIP).

**Figure 4:**
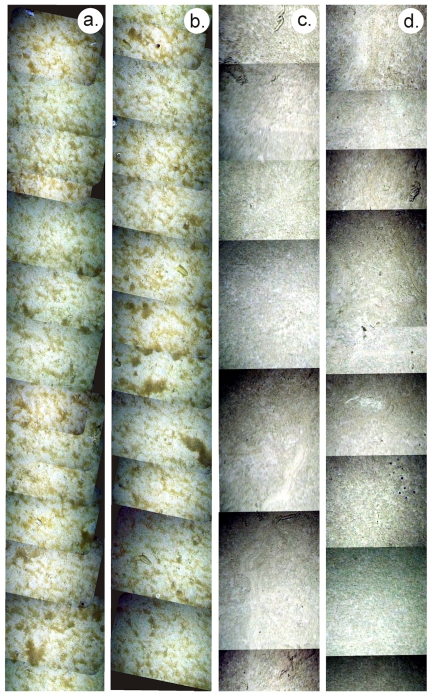
Seabed seafloor mosaic from WASP photographic images of: **a. & b.** the +Fe site, showing substantial cover of phytodetritus patches, which are clearly visible and appear green. **c. & d.** for comparison the HNLC site, where there is no phytodetritus but faecal casts are visible. In each case the swath width is c. 1.2 m and the total along track distance shown is approximately 2 m. (Total area illustrated c. 20 m^2^).

Sediment traps were deployed at the +Fe and HNLC sites at 3195 and 3183 m water depth (ca. 1000 m above the seafloor), respectively [12], [18]. The timing and temporal variability in flux was significantly different between the two sites (Fig. 2 a, b) [12]. At the +Fe site, east-southeast flows led to transport of exported flux from the Crozet bloom to the sediment trap. This extended over a period of at least 120 days originating from the spring/summer bloom in late 2004/early 2005. We deduce that the high-flux event towards the end of 2005 was derived from the onset of the late 2005 peak export flux to the deep sea. In contrast, downward flux at the HNLC site was focused in one very short event. It is not known if this represents a typical annual event, or one that occurs occasionally. For the purpose of this study we use the measured cumulative fluxes of organic carbon in the deep traps of 41.1 mmol m^−2^ (355 days) at the +Fe site, and of 14.1 mmol m^−2^ (352 days) at the HNLC site. At both sites, molar C/N ratios of organic matter reaching the sediment traps were close to the Redfield ratio (∼6.6), varying from 4.2 to 8.0. However, the chemical composition of the sediment trap material revealed substantial differences in organic matter quality between the two sites. Hence, fluxes (and concentrations) of labile lipids, including polyunsaturated fatty acids (PUFAs) and monounsaturated fatty acids (MUFAs), the presence of which suggest a source of high quality organic matter [32], [33], [34], were significantly higher (Friedman's test; n = 6 contiguous pairs, p = 0.014) at the +Fe site than at the HNLC site (Table 2; Fig. 3). PUFAs were below detection limits at the HNLC site. Photographs taken using the WASP system at the +Fe site in December 2005 confirm the presence of fresh phytodetritus at the seafloor (Fig. 4). We interpret this phytodetritus as originating from the late 2005 spring bloom event (Fig. 2a). Suspended POM (sPOM) collected using stand alone pump systems close to the seafloor at the +Fe site at that time had high chlorophyll and PUFA concentrations of 1.62 and 23.9 mg gC^−1^, respectively (cf. below detection and 5.6 mg gC^−1^, respectively at HNLC). The chlorophyll/phaeopigment ratio of surface sediments was also consistent with the presence of very fresh phytoplankton-derived material [35] (Table 2). WASP images showed no evidence of fresh phytodetritus at the seafloor at the HNLC site (Fig. 4).

**Table 2:** Total organic carbon (TOC), mean molar C/N, chlorophyll a and lipid concentrations (mg g^−1^ TOC) and ratio of chlorophyll a to pheopigments (Chl/P) in surface sediments (0–5 mm) and in sPOM at the Crozet islands abyssal locations, +Fe and HNLC at ∼4200 m.

	TOC^1^	C/N	Chl	Chl/P	Total Lipids	FA	Br-FA	MUFA	PUFA	ALK	Sterol	Other lipids^2^
**+Fe**												
*Sediment* *(0–5 mm)* 3	4.2 (0.3)	7.0 (0.7)	0.060 (0.007)	1.89	3.56 (1.08)	0.56 (0.11)	0.2 (0.04)	1.17 (0.24)	0.66 (0.49)	0.007 (0.003)	0.43 (0.11)	0.081 (0.035)
*sPOM at seafloor* 4	4.7 (0.5)	4.2	1.618	ND	85.7	20.6	0.5	21.6	23.9	2.8	15.2	0.8
**HNLC**												
*Sediment* *(0–5 mm)* 5	5.1 (1.1)	6.1 (0.85)	0.019 (0.014)	0.77	4.88 (2.54)	1.09 (0.49)	0.46 (0.29)	2.67 (1.67)	0.47 (0.48)	0.02 (0.007)	0.79 (0.68)	0.11 (0.05)
*sPOM at seafloor* 6	2.7 (1.1)	4.5	BD	ND	51.2	19.4	1.4	12.3	5.6	7.2	5.1	0.1

Concentrations are normalised to TOC to allow comparison of chlorophyll and lipid concentrations in sPOM and sediment samples.

TOC – Total organic carbon, Chl – Chlorophyll *a*, FAs - saturated fatty acids, Br-FAs – branched fatty acids; MUFAs – monunsaturated fatty acids; PUFAs – polyunsaturated fatty acids; ALKs – *n*-alkanols; US-ALKs – unsaturated alkanols; Other lipids include branched alkanols, tritepenoids, steroidal ketones, phytol derivatives and alkenones.

1 Units mg g^−1^ (dry sediment);

2 Other lipids include tritepenoids, phytol derivatives, alkenones and hydrocarbons.

3 n = 5, standard deviation in parentheses; except for chlorophyll, where n = 2, range in parentheses.

4 One SAP deployment, pumped 2138 L of water, 10 mab, units µg L^−1^ standard deviation of 5 replicate analyses of GFF filter;

5 n = 5 except for chlorophyll, where n = 4;

6 One SAP deployment, pumped 1517 L water, 60 mab, units µg L^−1^ standard deviation of 5 replicate analyses of GFF filter; BD Below Detection. ND Not Determined.

The total standing stock of invertebrate megafauna, in terms of biomass and abundance, mirrored the particulate organic carbon (POC) fluxes at the two sites (Table 3; Table S1; Fig. 2). It should be noted that we were able to collect four trawls at +Fe, but only two at HNLC because of severe technical difficulties [19]. Despite this, we have some confidence in our data because the video transects confirmed the significant difference in populations between the +Fe and HNLC sites. Biomass was ×3 greater at the +Fe site than at the HNLC site (15,251 and 5,083 g wet weight ha^−1^, respectively), while abundance was ∼×6 higher at +Fe *vs* the HNLC site (44,600 and 7,200 individuals ha^−1^, respectively). The close relationship between biomass and organic input has been observed in other abyssal ecosystems [36]. Abundance is a better measure of rate processes and ecosystem function [7], [37], [38]. The much higher abundances (Table 3; Table S1) suggest a higher rate of carbon cycling at the +Fe than the HNLC sites; this is reflected in the lower organic carbon contents of surface sediments at +Fe than HNLC (Table 2).

**Table 3:** The abundance and biomass (wet weight) of the dominant megafaunal invertebrates at abyssal sites around the Crozet Plateau.

Species Name	Taxon	Density (+Fe) (ind. ha^−1^) n = 4	Biomass (+Fe) (g ha^−1^) n = 4	Rank (+Fe) Abundance (Biomass)	Density (HNLC) (ind. ha^−1^) n = 2	Biomass (HNLC) (g ha^−1^) n = 2	Rank (HNLC) Abundance (Biomass)
*Peniagone crozeti*	Holothuroidea	***259.6***	***910.5***	1 (3)	***11.1***	***23.47***	
*Ophiura lienosa*	Ophiuroidea	194.7	53.43	2	162.3	64.25	1
*Amphioplus daleus*	Ophiuroidea	128	***35.69***	3	37.9	***6.615***	5
*Peniagone challengeri*	Holothuroidea	***69.2***	***137.1***	4	***5.6***	***9.894***	
*Ophiura irrorata loveni*	Ophiuroidea	41.3	38.53	5	18.7	17.61	
*Kolga nana*	Holothuroidea	0	0		17.4	3.276	
*Peniagone affinis*	Holothuroidea	***3.7***	***29.57***		***94.6***	***497.4***	3 (1)
*Peniagone willemoesi*	Holothuroidea	***1.8***	***4.544***		***95.6***	***134.2***	2 (3)
*Ophiotrema tertium*	Ophiuroidea	***0.04***	***7×10^−4^***		***61.1***	***7.633***	4
*Psychropotes longicauda*	Holothuroidea	***12.6***	***1195***	(1)	***2.5***	***105***	(5)
*Molpadiodemas aff. atlanticus*	Holothuroidea	***28.3***	***962.9***	(2)	***0***	***0***	
*Molpadiodemas morbillus*	Holothuroidea	***8.7***	***460.3***	(4)	***0***	***0***	
*Benthodytes sordida*	Holothuroidea	5.1	***308.7***	(5)	3.5	***131***	(4)
*Styracaster robustus*	Asteroidea	6.8	***52.11***		13.1	***230.1***	(2)

Bold italic numbers indicate significantly different populations in terms of abundance or biomass (p<0.05; ANOVA). Rankings (1–5) for the most abundant species and those having the highest biomass (parentheses) are also shown.

**Figure 5:**
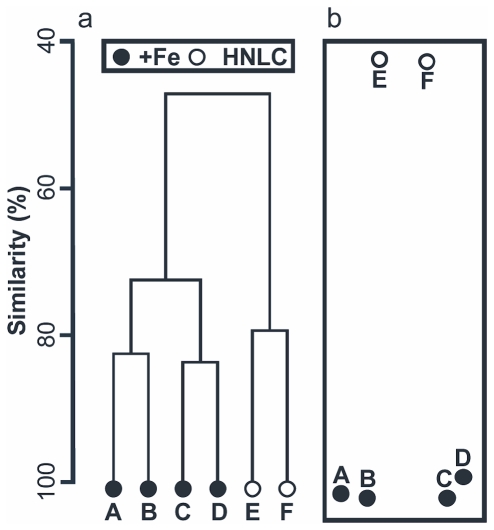
Variation in holothurian species composition between +Fe and HNLC sites. **a.** Dendrogram representation using group-average clustering. Based on trawl (A–F) catch data standardized to area fished, subject to log(x+1) transformation and assessed with Bray-Curtis similarity coefficient. **b.** Non-metric multidimensional scaling ordination plot (ordination stress = 0) for the same data.

Holothurians were the dominant megafaunal group at both sites accounting for between 70 and 89% of the total biomass. However, rather than the same species occurring at +Fe and HNLC sites, despite their close proximity, there were striking differences in species composition and dominance (Table 3; Figs. 5 a & b). Some species were common to the two sites, and occurred in similar abundances, such as the ophiuroids *Ophiura lienosa*, *Ophiura irrorata loveni* and *Amphioplus daleus*, but most megafaunal species showed very marked differences (Table 3, Table S1). Of the elpidiid holothurians *Kolga nana*, *Peniagone willemoesi* and *Peniagone affinis* dominated at the HNLC site, while closely related, but different, species, *Peniagone crozeti* [39] and *Peniagone challengeri* dominated at the +Fe site in all of the trawls.

## Discussion

**Figure 6:**
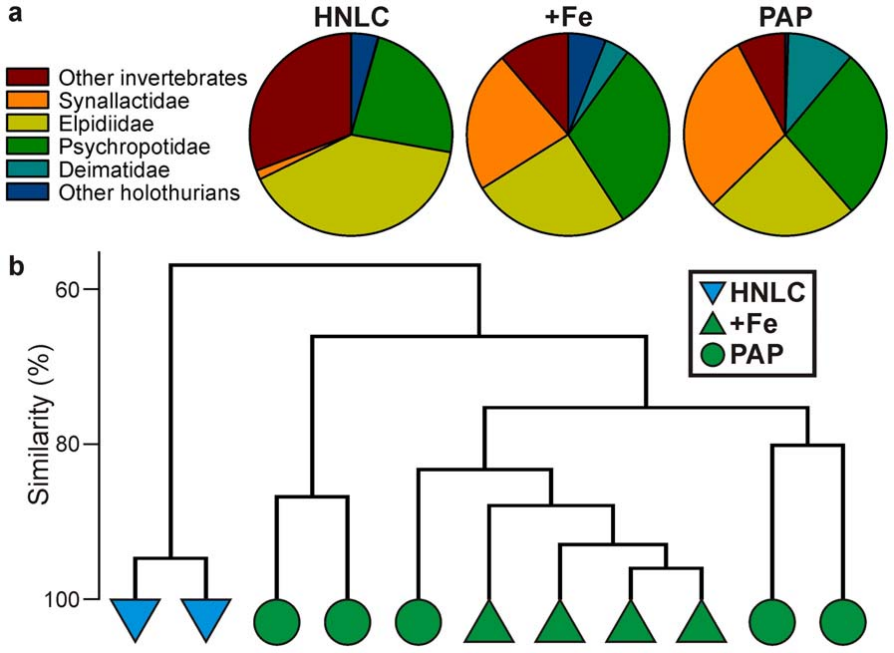
Fresh wet weight biomass composition of the megabenthos at Crozet (HNLC, +Fe) and the NE Atlantic Porcupine Abyssal Plain (PAP) sites. **A.** Average relative biomass of dominant holothurian families, combined ‘other holothurians’ and ‘other invertebrates’. **B.** Similarity of individual trawl catches based on the relative biomass of the same taxonomic groups (proportional biomass; Bray-Curtis similarity; group-average clustering).

If only one or two species had shown radical differences between the +Fe and HNLC sites then one might conclude that spatial patchiness was a key element in the observed differences. However, the marked contrast in many species suggests that environmental factors relating to organic matter supply are important in influencing species composition and ecosystem structure. In this context, the similarity in the composition of the dominant taxa at the +Fe site and at abyssal depths in the productive NE Atlantic, 16,000 km distant [40], [41], is remarkable. The relative contributions to the total biomass by different higher taxonomic groupings is almost identical in the two regions (Fig. 6). In addition, the same, or very similar, abundant holothurian species occur at widely separated eutrophic sites around the world [42].

This poses questions about what factors associated with organic supply are responsible for these broad-scale geographic patterns; is it the quantity, timing or quality of the POM flux or a combination of these factors? Our data suggest that the quality of POM arriving at the sea floor at +Fe was much higher than at HNLC. Phytoplankton communities were distinctly different at the +Fe and HNLC sites during the spring/summer 2004/2005, the former being dominated by *Phaeocystis* sp., the latter by large diatoms including *Fragilariopsis kerguelensis* [43]. The preserved planktonic assemblages in the deep-water sediment traps at the +Fe and HNLC sites were dominated by diatoms, but were also distinctly different, in that the neritic species *Eucampia antarctica* was abundant at +Fe, but completely absent at HNLC site [18]. These significant differences were reflected in the organic composition of the sinking POM collected in 1) the deep-water sediment traps (Fig. 3), 2) sPOM collected close to the seafloor and 3) surface sediments at the two sites (Table 2).

The greatly enhanced flux of labile compounds at +Fe *vs.* HNLC sites may be crucial in controlling species composition; this is supported indirectly by the differences in the biochemistry and fecundity of *Peniagone* species between sites. All the *Peniagone* species had a similar gametogenic biology and similar maximum egg size (ca. 500 µm diameter). The only difference was that *Peniagone crozeti* had a significantly higher gonad index (weight gonad/weight organism) at the +Fe site compared to all the other species, implying higher fecundity. Furthermore, the concentrations of carotenoids, which are important for echinoderm reproduction and enhance larval maturation and survival [44], [45], namely canthaxanthin, echinenone and β-carotene, were high in the ovaries of *Peniagone crozeti* at the +Fe site (Table S2). The supply of these pigments, which are believed to be derived from phytoplankton in overlying waters, and the ability of *P. crozeti*, in particular, to assimilate them, may be critical factors leading to the dominance of this species at +Fe. *Peniagone crozeti* was the single most abundant species around the Crozet Islands, occurring in great abundance in all of the trawls only at the +Fe site. It has never been recorded at any other locality. The distribution of *P. crozeti* appears to be restricted by the characteristics of the productivity regime in the overlying surface waters, pointing to the importance of organic input in regulating faunal distributions on the deep seafloor.

The timing and predictability of organic matter flux, including year-to-year consistency in flux, may also be important in structuring deep-sea benthic communities. Unfortunately, inter-annual sediment trap data are not available, but the HNLC site was notable for the prevalence of the holothurian species *Kolga nana*. *Kolga* has high fecundity and a small egg size indicative of ‘opportunistic’ species [22], [46]. It can occur episodically for short periods in high numbers [22], [47]. Its presence, together with the complete absence of species generally found in eutrophic settings, suggests that the character of the organic matter supply from year to year in HNLC regions may influence the composition of species in this region.

The quantity and composition of POM arriving at the seafloor in the Crozet region is influenced by the supply of iron to surface waters and this in turn controls the biomass and species composition of large invertebrate fauna at the seafloor. It is likely that enhanced carbon flux to the seafloor, *via* artificial OIF of the HNLC ocean, would change the deep-sea benthic biomass, species composition and dominance over an extended period of time. Furthermore, our study provides evidence that a combination of timing, quantity and quality of POM export strongly influence community structure of deep-sea benthic ecosystems.

## Supporting Information

Table S1 Abundance and biomass (wet weight) data for Echinoderm megafauna. Station numbers are taken from ref 19.
(DOCX)

Table S2 Pigment concentrations (µg gDW^−1^) in the ovaries of Peniagone spp. (n = 5 at each site) sampled at Crozet. 19′-but = 19′-butanoyloxyfucoxanthin; 19′-hex = 19′-hexanoyloxyfucoxanthin; Diadinox = diadinoxanthin; Allox = alloxanthin; Diatox = diatoxanthin; Zeax = zeaxanthin; Canthax = canthaxanthin; Echin = echinenone; β-carot = β-carotene. (Standard deviation in parentheses).
(DOCX)
