# Supplementary material for: The Effects of Natural Iron Fertilisation on Deep-Sea Ecology: The Crozet Plateau, Southern Indian Ocean
Source: PLoS One. 2011 Jun 14;6(6):e20697. doi: 10.1371/journal.pone.0020697 (PMC3114783; doi:10.1371/journal.pone.0020697)
Supplement: Table S1 — Abundance and biomass (wet weight) data for Echinoderm megafauna. Station numbers are taken from ref 19. (DOCX) [file pone.0020697.s001.docx]

| \| **Station** \| \| **+Fe 1**  **15773#8** \| **+Fe 2**  **15773#17** \| **+Fe 3**  **15573#23** \| **+Fe 4**  **15773#32** \| **HNLC 1**  **15775#13** \| **HNLC 2**  **15775#4** \| **+Fe 1**  **15773#8** \| **+Fe 2**  **15773#17** \| **+Fe 3**  **15773#23** \| **+Fe 4**  **15773#23** \| **HNLC 1**  **15775#13** \| **HNLC 2**  **15775#4** \| \| --- \| --- \| --- \| --- \| --- \| --- \| --- \| --- \| --- \| --- \| --- \| --- \| --- \| --- \| \| **Area Fished (ha)** \| \| 2.532 \| 6.833 \| 10.751 \| 4.714 \| 4.571 \| 4.316 \| 2.532 \| 6.833 \| 10.751 \| 4.714 \| 4.571 \| 4.316 \| \| **Taxon** \| **Species** \| **ABUNDANCE (ind. ha^-1^)** \| \| \| \| \| \| **BIOMASS (g ha^-1^)** \| \| \| \| \| \| \| Asteroidea \| *Eremicaster crassus* \| 9.48 \| 8.20 \|  \| 15.91 \| 1.53 \| 5.10 \| 17.89 \| 17.47 \|  \| 23.19 \| 6.08 \| 18.79 \| \| Asteroidea \| *Freyella sp.* \|  \|  \| 0.19 \|  \|  \|  \|  \|  \| 0.47 \|  \|  \|  \| \| Asteroidea \| *Hymenaster crucifer* \| 3.16 \| 4.39 \| 1.58 \| 2.33 \|  \|  \| 92.93 \| 126.84 \| 49.86 \| 58.02 \|  \|  \| \| Asteroidea \| *Hyphalaster australis* \| 1.58 \| 2.78 \| 8.74 \| 2.76 \| 1.97 \| 1.62 \| 3.79 \| 8.88 \| 17.91 \| 9.21 \| 12.40 \| 9.43 \| \| Asteroidea \| *Hyphalaster inermis* \|  \| 0.73 \|  \| 0.85 \|  \| 1.85 \|  \| 4.96 \|  \| 7.02 \|  \| 45.51 \| \| Asteroidea \| *Lonchotaster tartareus* \|  \| 0.44 \|  \|  \| 2.41 \| 6.02 \|  \| 17.88 \|  \|  \| 26.19 \| 109.48 \| \| Asteroidea \| *Lophaster ?antarcticus* \|  \| 0.44 \|  \|  \|  \|  \|  \| 0.44 \|  \|  \|  \|  \| \| Asteroidea \| *Paralophaster lorioli* \| 0.79 \| 0.29 \|  \| 0.21 \| 0.66 \| 0.93 \| 1.30 \| 0.94 \|  \| 0.11 \| 1.31 \| 1.25 \| \| Asteroidea \| *Porcellanaster ceruleus* \| 4.74 \| 1.02 \| 1.30 \| 7.85 \| 26.47 \| 30.35 \| 2.84 \| 0.45 \| 0.54 \| 3.80 \| 8.37 \| 9.61 \| \| Asteroidea \| *Pteraster ?hunteri* \|  \| 0.15 \|  \|  \|  \|  \|  \| 0.18 \|  \|  \|  \|  \| \| Asteroidea \| *Pteraster sp.* \| 1.18 \|  \|  \|  \|  \|  \| 0.95 \|  \|  \|  \|  \|  \| \| Asteroidea \| *Styracaster robustus* \| 5.53 \| 9.22 \| 4.28 \| 9.55 \| 13.56 \| 12.74 \| 63.23 \| 55.96 \| 29.88 \| 69.41 \| 214.31 \| 247.15 \| \| Ophiuroidea \| *Amphioplus antarctica* \|  \|  \|  \| 40.94 \|  \|  \|  \|  \|  \| 8.82 \|  \|  \| \| Ophiuroidea \| *Amphioplus daleus* \| 148.10 \| 99.08 \| 62.88 \| 289.78 \| 37.19 \| 38.69 \| 40.19 \| 30.37 \| 18.81 \| 69.82 \| 6.77 \| 6.46 \| \| Ophiuroidea \| *Amphiura studeri (antarctica)* \| 27.25 \| 13.46 \| 11.72 \| 0.00 \|  \|  \| 5.09 \| 2.90 \| 2.38 \|  \|  \|  \| \| Ophiuroidea \| *Ophiernus quadrispinus* \| 10.66 \| 12.59 \| 5.21 \|  \|  \| 3.94 \| 5.61 \| 5.69 \| 2.28 \|  \|  \| 2.37 \| \| Ophiuroidea \| *Ophiolepis ?scissa* \|  \| 2.93 \|  \|  \|  \| 1.16 \|  \| 0.34 \|  \|  \|  \| 0.06 \| \| Ophiuroidea \| *Ophiotrema tertium* \|  \| 0.15 \|  \|  \| 31.72 \| 116.77 \|  \| 0.01 \|  \|  \| 3.65 \| 15.02 \| \| Ophiuroidea \| *Ophiuria irrorata loveni* \| 30.81 \| 49.17 \| 28.18 \| 67.88 \| 11.38 \| 30.35 \| 29.86 \| 47.08 \| 24.19 \| 64.35 \| 10.66 \| 28.71 \| \| Ophiuroidea \| *Ophiuria lienosa* \| 277.25 \| 146.06 \| 69.67 \| 506.15 \| 147.01 \| 179.10 \| 92.69 \| 40.42 \| 17.50 \| 121.20 \| 57.68 \| 71.57 \| \| Ophiuroidea \| *Opiacantha cosmica* \| 11.45 \| 5.56 \| 4.09 \| 15.49 \| 19.25 \| 47.73 \| 4.03 \| 3.53 \| 2.67 \| 8.40 \| 7.66 \| 30.63 \| \| Echinoidea \| *Echinoidea spp fragments total* \|  \|  \|  \|  \|  \|  \| 12.16 \| 0.44 \| 1.79 \|  \| 4.44 \| 61.58 \| \| Echinoidea \| *Kamptosoma abyssale* \|  \| 0.15 \| 0.37 \| 0.42 \| 0.66 \| 2.09 \|  \| 0.70 \| 2.40 \| 2.08 \| 2.63 \| 7.97 \| \| Holothuroidea \| *Abyssocucumis abyssorum* \| 26.86 \| 23.71 \| 0.37 \| 41.79 \| 5.91 \| 4.40 \| 316.59 \| 170.38 \| 0.85 \| 413.19 \| 46.90 \| 34.66 \| \| Holothuroidea \| *Amperima robusta* \| 15.01 \| 40.68 \| 20.18 \| 32.03 \| 4.59 \| 3.71 \| 112.84 \| 184.00 \| 173.32 \| 179.55 \| 3.04 \| 1.30 \| \| Holothuroidea \| *Bathyplotes gourdoni* \| 1.18 \|  \| 0.28 \| 0.21 \|  \|  \| 2.05 \|  \| 0.78 \| 0.04 \|  \|  \| \| Holothuroidea \| *Bathyplotes natans* \|  \| 0.15 \|  \| 0.21 \|  \|  \|  \| 0.69 \|  \| 0.36 \|  \|  \| \| Holothuroidea \| *Benthodytes abyssicola* \| 3.16 \| 4.98 \| 6.42 \| 6.36 \| 2.84 \| 4.17 \| 197.87 \| 281.69 \| 369.56 \| 440.64 \| 143.82 \| 119.37 \| \| Holothuroidea \| *Benthodytes sanguinolenta* \| 11.45 \| 14.93 \| 12.37 \| 16.97 \| 3.72 \| 2.55 \| 320.26 \| 230.51 \| 326.57 \| 262.71 \| 68.30 \| 66.52 \| \| Holothuroidea \| *Benthodytes wolffi* \| 1.18 \| 0.73 \| 0.09 \| 4.03 \| 5.69 \| 3.01 \| 54.11 \| 12.59 \| 0.17 \| 69.52 \| 33.41 \| 34.92 \| \| Holothuroidea \| *Ellipinion papillosum* \| 1.97 \| 0.29 \|  \| 1.91 \| 0.22 \| 0.93 \| 14.89 \| 0.75 \|  \| 12.22 \| 0.04 \| 0.58 \| \| Holothuroidea \| *Elpidia ?theeli* \|  \|  \|  \|  \| 11.81 \| 7.18 \|  \|  \|  \|  \| 1.11 \| 0.39 \| \| Holothuroidea \| *Enypniastes exigua* \| 0.39 \|  \|  \|  \|  \|  \| 1.07 \|  \|  \|  \|  \|  \| \| Holothuroidea \| *Gebrukothuria profundus* \|  \|  \| 0.09 \|  \|  \|  \|  \|  \| 14.00 \|  \|  \|  \| \| Holothuroidea \| *Kolga nana* \|  \|  \|  \|  \| 2.84 \| 338.74 \|  \|  \|  \|  \| 0.23 \| 17.28 \| \| Holothuroidea \| *Laetmogone wyvillethomsoni* \| 2.76 \| 1.32 \| 1.21 \| 1.91 \|  \|  \| 27.13 \| 12.59 \| 27.17 \| 8.95 \|  \|  \| \| Holothuroidea \| *Mesothuria edwardensis* \|  \|  \|  \|  \| 0.44 \|  \|  \|  \|  \|  \| 4.92 \|  \| \| Holothuroidea \| *Molpadia blakei* \| 0.79 \| 3.07 \| 1.40 \| 4.24 \| 0.88 \| 0.70 \| 42.46 \| 47.56 \| 60.27 \| 89.41 \| 11.31 \| 2.09 \| \| Holothuroidea \| *Molpadiodemas aff atlanticus* \| 14.22 \| 29.42 \| 26.79 \| 56.43 \|  \|  \| 649.13 \| 624.02 \| 1125.12 \| 1885.00 \|  \|  \| \| Holothuroidea \| *Molpadiodemas crinitus* \| 0.79 \| 0.88 \| 0.56 \| 1.91 \|  \|  \| 28.59 \| 18.10 \| 13.60 \| 25.69 \|  \|  \| \| Holothuroidea \| *Molpadiodemas involutus* \|  \|  \| 0.19 \|  \|  \|  \|  \|  \| 0.13 \|  \|  \|  \| \| Holothuroidea \| *Molpadiodemas morbillus* \| 4.34 \| 9.81 \| 7.53 \| 16.76 \|  \|  \| 296.25 \| 388.10 \| 453.06 \| 861.46 \|  \|  \| \| Holothuroidea \| *Molpadiodemas translucens* \| 0.79 \| 0.73 \| 0.28 \| 0.85 \| 0.22 \| 0.46 \| 3.55 \| 3.48 \| 1.36 \| 3.84 \| 0.24 \| 3.75 \| \| Holothuroidea \| *Molpadiodemas villosus* \|  \| 0.15 \| 0.65 \|  \|  \|  \|  \| 0.25 \| 2.98 \|  \|  \|  \| \| Holothuroidea \| *Oneirophanta mutabilis* \| 16.98 \| 21.95 \| 17.86 \| 35.43 \| 0.66 \| 4.17 \| 212.40 \| 259.92 \| 247.74 \| 466.84 \| 0.24 \| 17.68 \| \| Holothuroidea \| *Paelopatides confundens* \|  \|  \|  \| 0.85 \|  \|  \|  \|  \|  \| 7.68 \|  \|  \| \| Holothuroidea \| *Paelopatides grisea* \| 2.76 \| 2.20 \| 3.53 \| 7.85 \| 1.09 \| 0.23 \| 32.94 \| 39.18 \| 117.56 \| 152.61 \| 9.69 \| 3.92 \| \| Holothuroidea \| *Paroriza sp. A* \|  \|  \| 0.09 \|  \|  \|  \|  \|  \| 21.04 \|  \|  \|  \| \| Holothuroidea \| *Paroriza sp. B* \|  \|  \|  \| 0.21 \|  \|  \|  \|  \|  \| 3.63 \|  \|  \| \| Holothuroidea \| *Peniagone crozeti* \| 280.41 \| 207.38 \| 165.38 \| 471.79 \| 18.38 \| 6.49 \| 1353.44 \| 553.23 \| 552.97 \| 1658.78 \| 40.47 \| 13.44 \| \| Holothuroidea \| *Peniagone affinis* \| 3.95 \| 3.51 \| 2.51 \| 5.09 \| 78.98 \| 113.30 \| 49.76 \| 24.62 \| 10.52 \| 57.28 \| 365.35 \| 677.07 \| \| Holothuroidea \| *Peniagone challengeri* \| 65.96 \| 65.86 \| 59.53 \| 88.46 \| 6.13 \| 5.10 \| 188.47 \| 110.89 \| 119.32 \| 141.79 \| 14.37 \| 6.72 \| \| Holothuroidea \| *Peniagone diaphana* \| 0.39 \|  \| 0.09 \|  \| 1.09 \| 0.46 \| 0.51 \|  \| 0.10 \|  \| 0.20 \| 0.23 \| \| Holothuroidea \| *Peniagone elongata* \| 9.87 \| 9.66 \| 17.02 \| 14.85 \| 66.94 \| 52.83 \| 42.30 \| 29.97 \| 47.56 \| 44.25 \| 85.04 \| 65.66 \| \| Holothuroidea \| *Peniagone gracilis* \| 0.79 \| 0.59 \| 1.02 \| 0.85 \| 14.00 \| 6.95 \| 2.69 \| 0.44 \| 1.58 \| 2.50 \| 8.25 \| 2.72 \| \| Holothuroidea \| *Peniagone horrifer* \| 4.74 \| 0.44 \| 0.84 \| 0.42 \| 20.56 \| 8.11 \| 11.33 \| 0.69 \| 1.33 \| 0.64 \| 17.65 \| 9.22 \| \| Holothuroidea \| *Peniagone purpurea* \|  \|  \|  \|  \| 12.69 \| 5.10 \|  \|  \|  \|  \| 4.38 \| 2.32 \| \| Holothuroidea \| *Peniagone vitrea* \| 0.79 \| 0.44 \| 0.28 \| 1.06 \| 8.09 \| 12.97 \| 1.03 \| 0.37 \| 0.32 \| 0.87 \| 7.74 \| 9.13 \| \| Holothuroidea \| *Peniagone willemoesi* \| 1.97 \| 1.90 \| 1.77 \| 1.70 \| 99.32 \| 91.98 \| 5.33 \| 4.96 \| 5.24 \| 3.01 \| 157.97 \| 113.99 \| \| Holothuroidea \| *Protankyra brychia* \| 2.76 \| 2.34 \| 0.74 \| 4.03 \|  \| 4.17 \| 8.45 \| 4.05 \| 1.55 \| 10.27 \|  \| 15.18 \| \| Holothuroidea \| *Pseudostichopus echinatus* \|  \|  \| 0.09 \|  \|  \|  \|  \|  \| 0.10 \|  \|  \|  \| \| Holothuroidea \| *Pseudostichopus mollis* \| 0.79 \| 1.61 \| 1.67 \| 1.70 \| 0.22 \| 0.46 \| 2.73 \| 4.20 \| 4.64 \| 4.54 \| 2.03 \| 5.68 \| \| Holothuroidea \| *Pseudostichopus peripatus* \| 5.13 \| 0.88 \| 3.35 \|  \| 1.97 \|  \| 3.79 \| 1.26 \| 1.93 \|  \| 0.97 \|  \| \| Holothuroidea \| *Pseudostichopus sp.* \|  \| 0.15 \| 0.19 \|  \|  \|  \|  \| 0.28 \| 0.27 \|  \|  \|  \| \| Holothuroidea \| *Psychrelpidia verrucosa* \| 1.18 \| 0.73 \| 0.65 \| 0.21 \| 1.75 \| 0.23 \| 3.95 \| 0.66 \| 0.55 \| 0.19 \| 2.87 \| 0.14 \| \| Holothuroidea \| *Psychroplanes convexa* \|  \|  \| 0.09 \|  \| 2.19 \| 3.01 \|  \|  \| 0.39 \|  \| 1.44 \| 1.99 \| \| Holothuroidea \| *Psychropotes scotiae* \| 2.76 \| 0.59 \| 1.40 \| 0.64 \| 0.22 \| 3.48 \| 110.78 \| 22.22 \| 122.22 \| 36.61 \| 0.02 \| 15.75 \| \| Holothuroidea \| *Psychropotes longicauda* \| 8.29 \| 14.20 \| 10.70 \| 19.52 \| 1.53 \| 3.94 \| 1137.68 \| 1098.58 \| 1140.28 \| 1568.48 \| 115.07 \| 95.76 \| \| Holothuroidea \| *Psychrotrephes discoveryi* \|  \| 0.59 \| 0.47 \| 1.06 \| 0.88 \| 1.16 \|  \| 3.76 \| 33.79 \| 27.13 \| 4.68 \| 4.36 \| \| Holothuroidea \| *Scotoplanes globosa* \| 1.97 \| 2.34 \| 0.84 \| 2.33 \|  \|  \| 94.31 \| 46.45 \| 28.43 \| 98.79 \|  \|  \| \| Holothuroidea \| *Ypsilothuria sp.* \|  \| 0.15 \| 0.47 \| 0.42 \|  \|  \|  \| 0.31 \| 0.76 \| 0.57 \|  \|  \| \| Holothuroidea \| *Zygothuria lactea* \|  \|  \|  \|  \| 0.44 \|  \|  \|  \|  \|  \| 0.35 \|  \| |  |  |  |  |  |  |  |  |  |  |  |  |  |  |  |  |
| --- | --- | --- | --- | --- | --- | --- | --- | --- | --- | --- | --- | --- | --- | --- | --- | --- | --- | --- | --- | --- | --- | --- | --- | --- | --- | --- | --- | --- | --- | --- | --- | --- | --- | --- | --- | --- | --- | --- | --- | --- | --- | --- | --- | --- | --- | --- | --- | --- | --- | --- | --- | --- | --- | --- | --- | --- | --- | --- | --- | --- | --- | --- | --- | --- | --- | --- | --- | --- | --- | --- | --- | --- | --- | --- | --- | --- | --- | --- | --- | --- | --- | --- | --- | --- | --- | --- | --- | --- | --- | --- | --- | --- | --- | --- | --- | --- | --- | --- | --- | --- | --- | --- | --- | --- | --- | --- | --- | --- | --- | --- | --- | --- | --- | --- | --- | --- | --- | --- | --- | --- | --- | --- | --- | --- | --- | --- | --- | --- | --- | --- | --- | --- | --- | --- | --- | --- | --- | --- | --- | --- | --- | --- | --- | --- | --- | --- | --- | --- | --- | --- | --- | --- | --- | --- | --- | --- | --- | --- | --- | --- | --- | --- | --- | --- | --- | --- | --- | --- | --- | --- | --- | --- | --- | --- | --- | --- | --- | --- | --- | --- | --- | --- | --- | --- | --- | --- | --- | --- | --- | --- | --- | --- | --- | --- | --- | --- | --- | --- | --- | --- | --- | --- | --- | --- | --- | --- | --- | --- | --- | --- | --- | --- | --- | --- | --- | --- | --- | --- | --- | --- | --- | --- | --- | --- | --- | --- | --- | --- | --- | --- | --- | --- | --- | --- | --- | --- | --- | --- | --- | --- | --- | --- | --- | --- | --- | --- | --- | --- | --- | --- | --- | --- | --- | --- | --- | --- | --- | --- | --- | --- | --- | --- | --- | --- | --- | --- | --- | --- | --- | --- | --- | --- | --- | --- | --- | --- | --- | --- | --- | --- | --- | --- | --- | --- | --- | --- | --- | --- | --- | --- | --- | --- | --- | --- | --- | --- | --- | --- | --- | --- | --- | --- | --- | --- | --- | --- | --- | --- | --- | --- | --- | --- | --- | --- | --- | --- | --- | --- | --- | --- | --- | --- | --- | --- | --- | --- | --- | --- | --- | --- | --- | --- | --- | --- | --- | --- | --- | --- | --- | --- | --- | --- | --- | --- | --- | --- | --- | --- | --- | --- | --- | --- | --- | --- | --- | --- | --- | --- | --- | --- | --- | --- | --- | --- | --- | --- | --- | --- | --- | --- | --- | --- | --- | --- | --- | --- | --- | --- | --- | --- | --- | --- | --- | --- | --- | --- | --- | --- | --- | --- | --- | --- | --- | --- | --- | --- | --- | --- | --- | --- | --- | --- | --- | --- | --- | --- | --- | --- | --- | --- | --- | --- | --- | --- | --- | --- | --- | --- | --- | --- | --- | --- | --- | --- | --- | --- | --- | --- | --- | --- | --- | --- | --- | --- | --- | --- | --- | --- | --- | --- | --- | --- | --- | --- | --- | --- | --- | --- | --- | --- | --- | --- | --- | --- | --- | --- | --- | --- | --- | --- | --- | --- | --- | --- | --- | --- | --- | --- | --- | --- | --- | --- | --- | --- | --- | --- | --- | --- | --- | --- | --- | --- | --- | --- | --- | --- | --- | --- | --- | --- | --- | --- | --- | --- | --- | --- | --- | --- | --- | --- | --- | --- | --- | --- | --- | --- | --- | --- | --- | --- | --- | --- | --- | --- | --- | --- | --- | --- | --- | --- | --- | --- | --- | --- | --- | --- | --- | --- | --- | --- | --- | --- | --- | --- | --- | --- | --- | --- | --- | --- | --- | --- | --- | --- | --- | --- | --- | --- | --- | --- | --- | --- | --- | --- | --- | --- | --- | --- | --- | --- | --- | --- | --- | --- | --- | --- | --- | --- | --- | --- | --- | --- | --- | --- | --- | --- | --- | --- | --- | --- | --- | --- | --- | --- | --- | --- | --- | --- | --- | --- | --- | --- | --- | --- | --- | --- | --- | --- | --- | --- | --- | --- | --- | --- | --- | --- | --- | --- | --- | --- | --- | --- | --- | --- | --- | --- | --- | --- | --- | --- | --- | --- | --- | --- | --- | --- | --- | --- | --- | --- | --- | --- | --- | --- | --- | --- | --- | --- | --- | --- | --- | --- | --- | --- | --- | --- | --- | --- | --- | --- | --- | --- | --- | --- | --- | --- | --- | --- | --- | --- | --- | --- | --- | --- | --- | --- | --- | --- | --- | --- | --- | --- | --- | --- | --- | --- | --- | --- | --- | --- | --- | --- | --- | --- | --- | --- | --- | --- | --- | --- | --- | --- | --- | --- | --- | --- | --- | --- | --- | --- | --- | --- | --- | --- | --- | --- | --- | --- | --- | --- | --- | --- | --- | --- | --- | --- | --- | --- | --- | --- | --- | --- | --- | --- | --- | --- | --- | --- | --- | --- | --- | --- | --- | --- | --- | --- | --- | --- | --- | --- | --- | --- | --- | --- | --- | --- | --- | --- | --- | --- | --- | --- | --- | --- | --- | --- | --- | --- | --- | --- | --- | --- | --- | --- | --- | --- | --- | --- | --- | --- | --- | --- | --- | --- | --- | --- | --- | --- | --- | --- | --- | --- | --- | --- | --- | --- | --- | --- | --- | --- | --- | --- | --- | --- | --- | --- | --- | --- | --- | --- | --- | --- | --- | --- | --- | --- | --- | --- | --- | --- | --- | --- | --- | --- | --- | --- | --- | --- | --- | --- | --- | --- | --- | --- | --- | --- | --- | --- | --- | --- | --- | --- | --- | --- | --- | --- | --- | --- | --- | --- | --- | --- | --- | --- | --- | --- | --- | --- | --- | --- | --- | --- | --- | --- | --- | --- | --- | --- | --- | --- | --- | --- | --- | --- | --- | --- | --- | --- | --- | --- | --- | --- | --- | --- | --- | --- | --- | --- | --- | --- | --- | --- | --- | --- | --- | --- | --- | --- | --- | --- | --- | --- | --- | --- | --- | --- | --- | --- | --- | --- | --- | --- | --- | --- | --- | --- | --- | --- | --- | --- | --- | --- | --- | --- | --- | --- | --- | --- | --- | --- | --- | --- | --- | --- | --- | --- | --- | --- | --- | --- | --- | --- | --- | --- | --- | --- | --- | --- | --- | --- | --- | --- | --- | --- | --- | --- | --- | --- | --- | --- | --- | --- | --- | --- | --- | --- | --- | --- | --- | --- | --- | --- | --- | --- | --- | --- | --- | --- | --- | --- | --- | --- | --- | --- | --- | --- | --- | --- | --- | --- | --- | --- | --- | --- | --- | --- | --- | --- | --- | --- | --- | --- | --- | --- | --- | --- | --- | --- | --- | --- | --- | --- | --- | --- | --- | --- | --- | --- | --- | --- | --- | --- | --- | --- | --- | --- | --- | --- | --- | --- | --- | --- | --- | --- | --- | --- | --- | --- | --- | --- | --- | --- | --- | --- | --- | --- | --- | --- | --- | --- | --- | --- | --- | --- | --- | --- | --- | --- | --- | --- | --- | --- | --- | --- | --- | --- | --- | --- | --- | --- | --- | --- | --- | --- | --- | --- |
