# Supplementary material for: The Effects of Natural Iron Fertilisation on Deep-Sea Ecology: The Crozet Plateau, Southern Indian Ocean
Source: PLoS One. 2011 Jun 14;6(6):e20697. doi: 10.1371/journal.pone.0020697 (PMC3114783; doi:10.1371/journal.pone.0020697)
Supplement: Table S2 — Pigment concentrations (µg gDW−1) in the ovaries of Peniagone spp. (n = 5 at each site) sampled at Crozet. 19′-but = 19′-butanoyloxyfucoxanthin; 19′-hex = 19′-hexanoyloxyfucoxanthin; Diadinox = diadinoxanthin; Allox = alloxanthin; Diatox = diatoxanthin; Zeax = zeaxanthin; Canthax = canthaxanthin; Echin = echinenone; β-carot = β-carotene. (Standard deviation in parentheses). (DOCX) [file pone.0020697.s002.docx]

| **Pigment** | **19’-but** | **19’-hex** | **Diadinox** | **Allox** | **Diatox** | **Zeax** | **Canthax** | **Echin** | **β-Carot** |
| --- | --- | --- | --- | --- | --- | --- | --- | --- | --- |
| **+Fe** | 1.11  (1.12) | 0.62  (0.56) | 0.21  (0.24) | 1.19  (0.98) | 3.19  (3.13) | 11.89  (10.75) | 1.43  (1.01) | 8.62  (6.35) | 19.46 (15.29) |
| **HNLC** | 0 | 0.80  (0.76) | 0.24  (0.33) | 0.23  (0.44) | 0.47  (0.30) | 0.90  (0.88) | 0.63  (0.38) | 2.64  (1.64) | 3.08  (2.56) |

|  |  |  |  |  |  |  |  |  |  |  |  |  |  |  |  |  |
| --- | --- | --- | --- | --- | --- | --- | --- | --- | --- | --- | --- | --- | --- | --- | --- | --- |
|  |  |  |  |  |  |  |  |  |  |  |  |  |  |  |  |  |
|  |  |  |  |  |  |  |  |  |  |  |  |  |  |  |  |  |
|  |  |  |  |  |  |  |  |  |  |  |  |  |  |  |  |  |
|  |  |  |  |  |  |  |  |  |  |  |  |  |  |  |  |  |
|  |  |  |  |  |  |  |  |  |  |  |  |  |  |  |  |  |
